# Supplementary material for: Fabrication of robust and cost-efficient Hoffmann-type MOF sensors for room temperature ammonia detection
Source: Nat Commun. 2023 Nov 9;14:7261. doi: 10.1038/s41467-023-42959-z (PMC10636145; doi:10.1038/s41467-023-42959-z)
Supplement: Supplementary file 1 — Supplementary Information [file 41467_2023_42959_MOESM1_ESM.pdf]

Supporting Information

Fabrication of robust and cost-efficient Hoffmann-type MOF sensors for room temperature ammonia detection

Sa Wang,[1,2] Yu Fu,[5] Ting Wang,[1,2] Wansheng Liu,[1,2] Jian Wang,[1,3] Peng Zhao[2], Heping Ma,[5] Yao Chen,[1,3] Peng Cheng,[1,2,4] & Zhenjie Zhang[1,2,4*]

[1]College of Chemistry, State Key Laboratory of Medicinal Chemical Biology, Nankai University, Tianjin 300071

[2]Key Laboratory of Advanced Energy Materials Chemistry, Ministry of Education, Nankai University, Tianjin 300071 (China)

[3]College of Pharmacy, Nankai University, Tianjin 300071 (China)

[4]Renewable energy conversion and storage center, Nankai University, Tianjin 300071 (China)

[5]School of Chemical Engineering and Technology, Xi'an Jiaotong University, Xi'an 710049, P. R. China.

*Corresponding author.

Email: zhangzhenjie@nankai.edu.cn

Supplementary Figures.

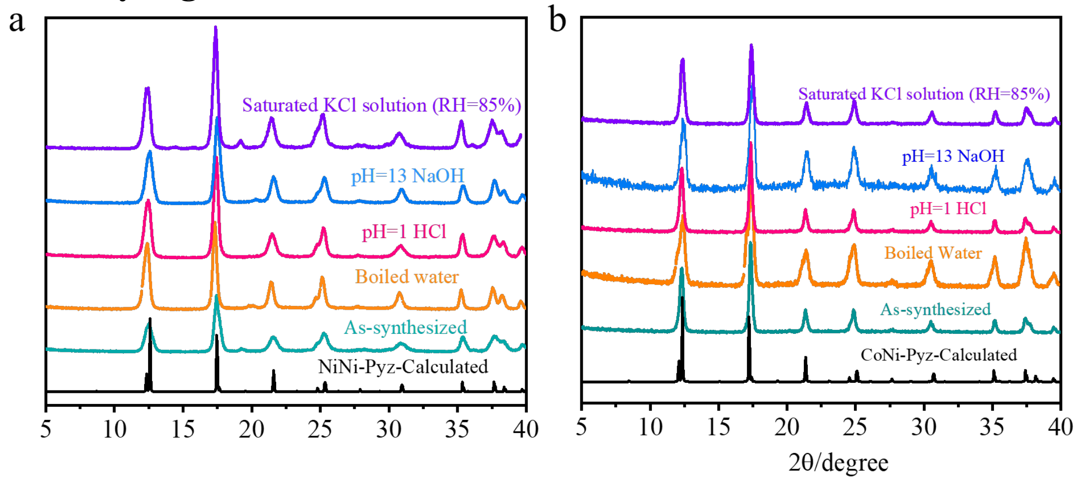

Supplementary Fig. 1. PXRD patterns. (a) NiNi-Pyz and (b) CoNi-Pyz after various treating conditions.

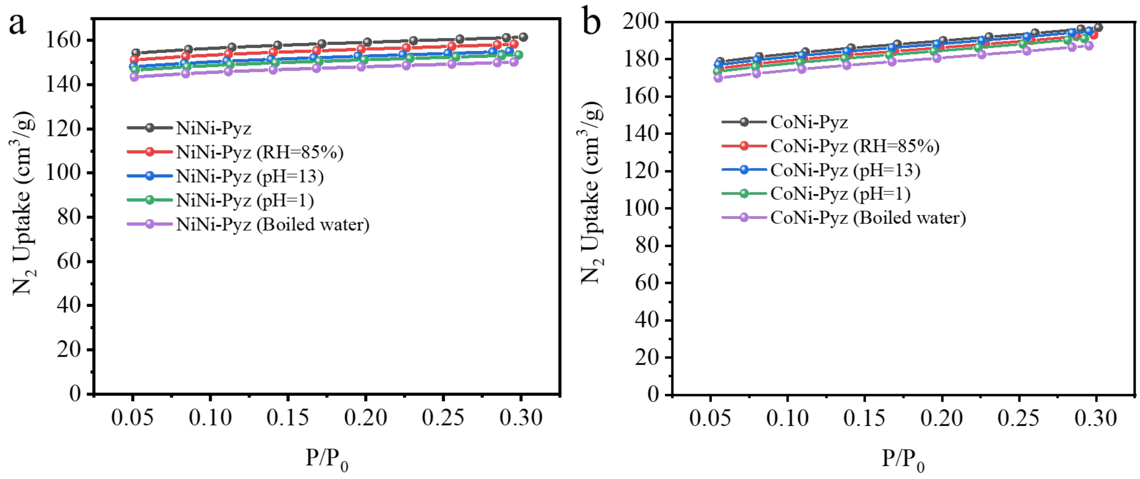

Supplementary Fig. 2. BET of MNi-Pyz. (a) NiNi-Pyz and (b) CoNi-Pyz after various treatments.

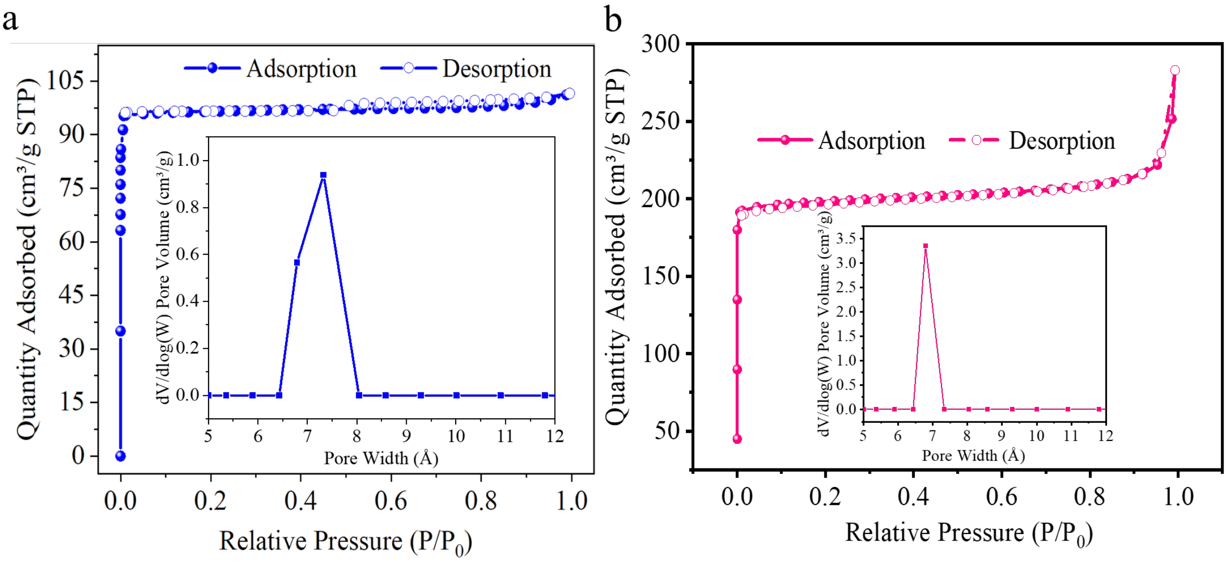

Supplementary Fig. 3. N_2-all characterization of MNi-Pyz. The adsorption-desorption isotherms of N_2 at 77 K, and the pore size distribution (inset) for (a) NiNi-Pyz and (b) CoNi-Pyz.

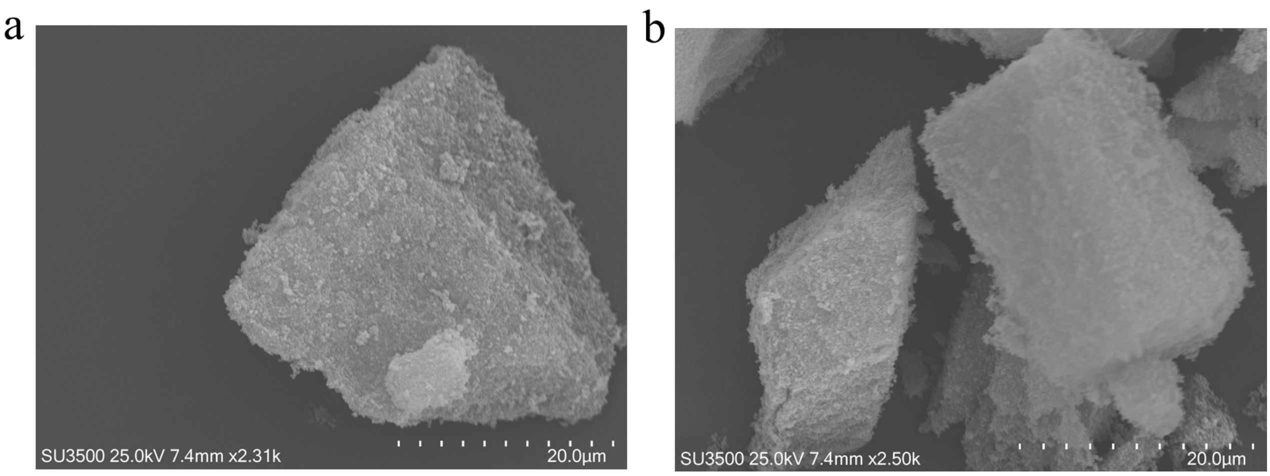

Supplementary Fig. 4. SEM characterization of MNi-Pyz. (a) NiNi-Pyz and (b) CoNi-Pyz.

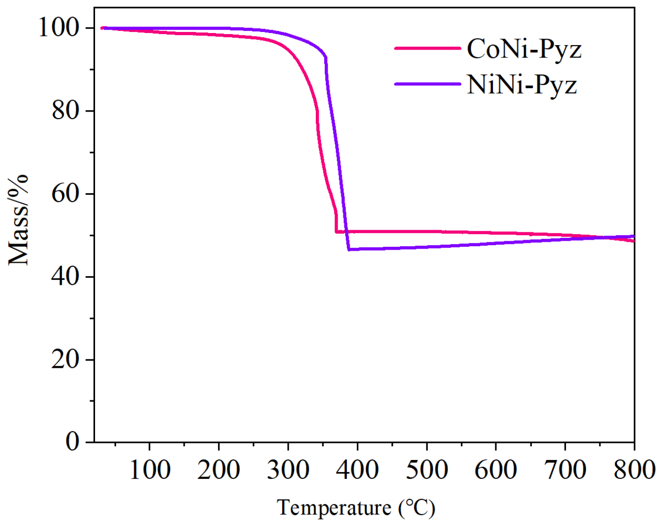

Supplementary Fig. 5. TGA characterization of MNi-Pyz. (a) NiNi-Pyz and (b) CoNi-Pyz.

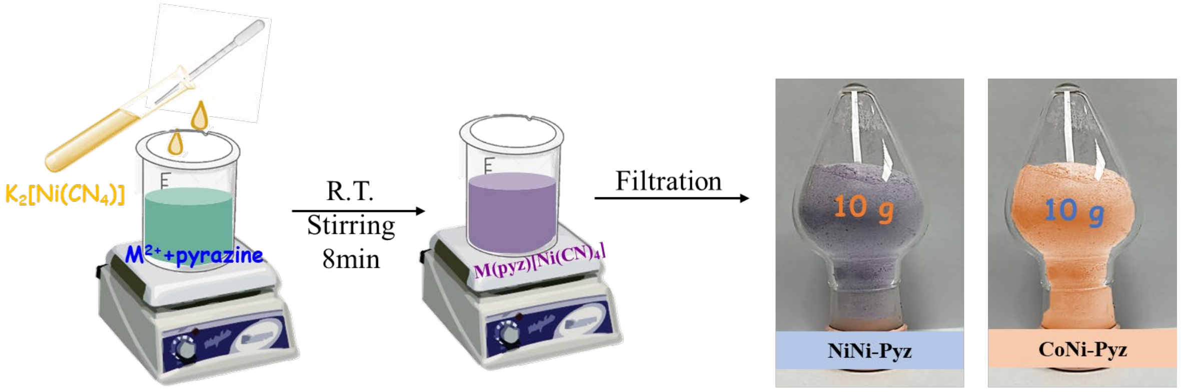

Supplementary Fig. 6. Schematic diagram of rapid synthesis. (a) NiNi-Pyz and (b) CoNi-Pyz.

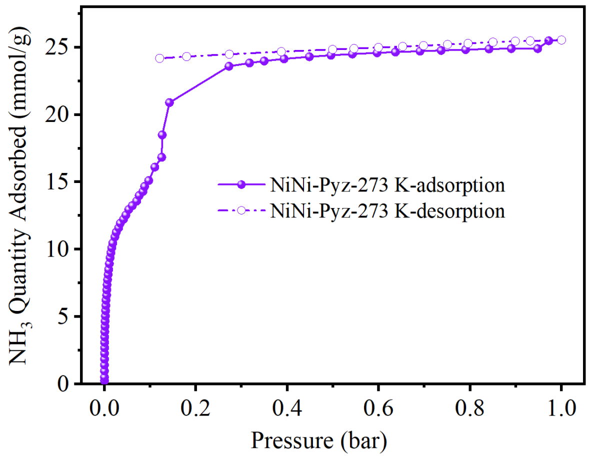

Supplementary Fig. 7. NH_3 adsorption of NiNi-Pyz. NH_3 adsorption-desorption isotherms of NiNi-Pyz at 298 K.

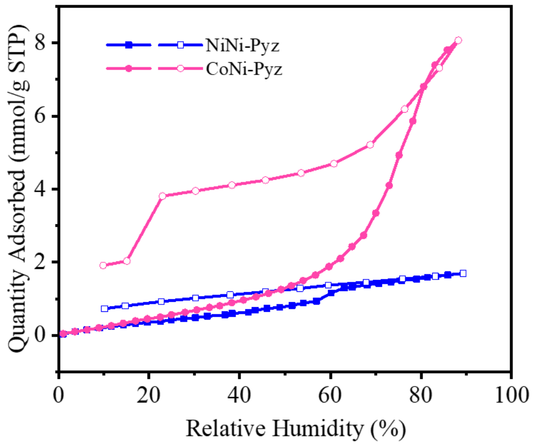

Supplementary Fig. 8. H_2O adsorption of MNi-Pyz. Water vapor adsorption of NiNi-Pyz and CoNi-Pyz at 298 K.

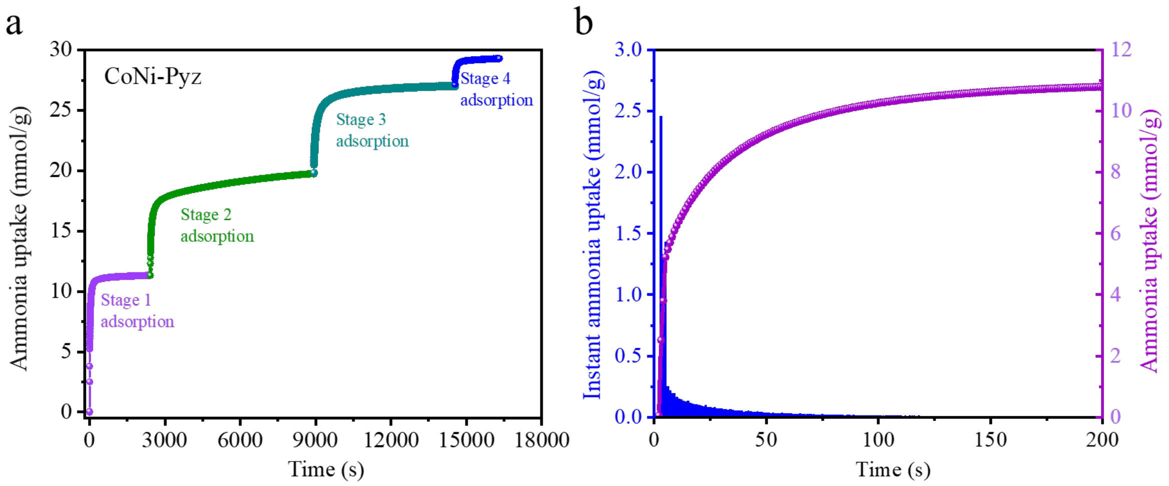

Supplementary Fig. 9. Dynamic test for CoNi-Pyz. (a) Kinetic adsorption curves and (b) preferential stage 1 adsorption curve for CoNi-Pyz to NH_3 at 298 K.

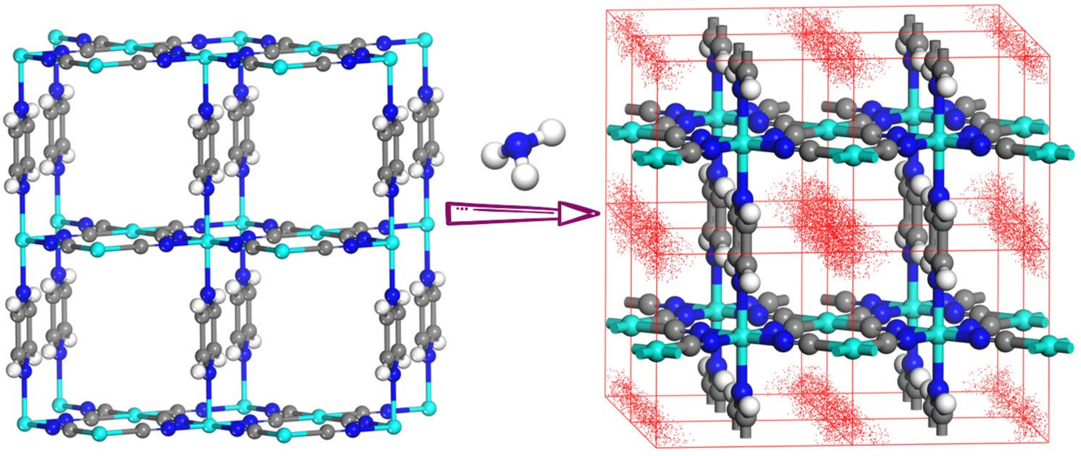

Supplementary Fig. 10. GCMC simulated NH_3 adsorption field distribution of MNi-Pyz. Total adsorption field of MNi-Pyz.

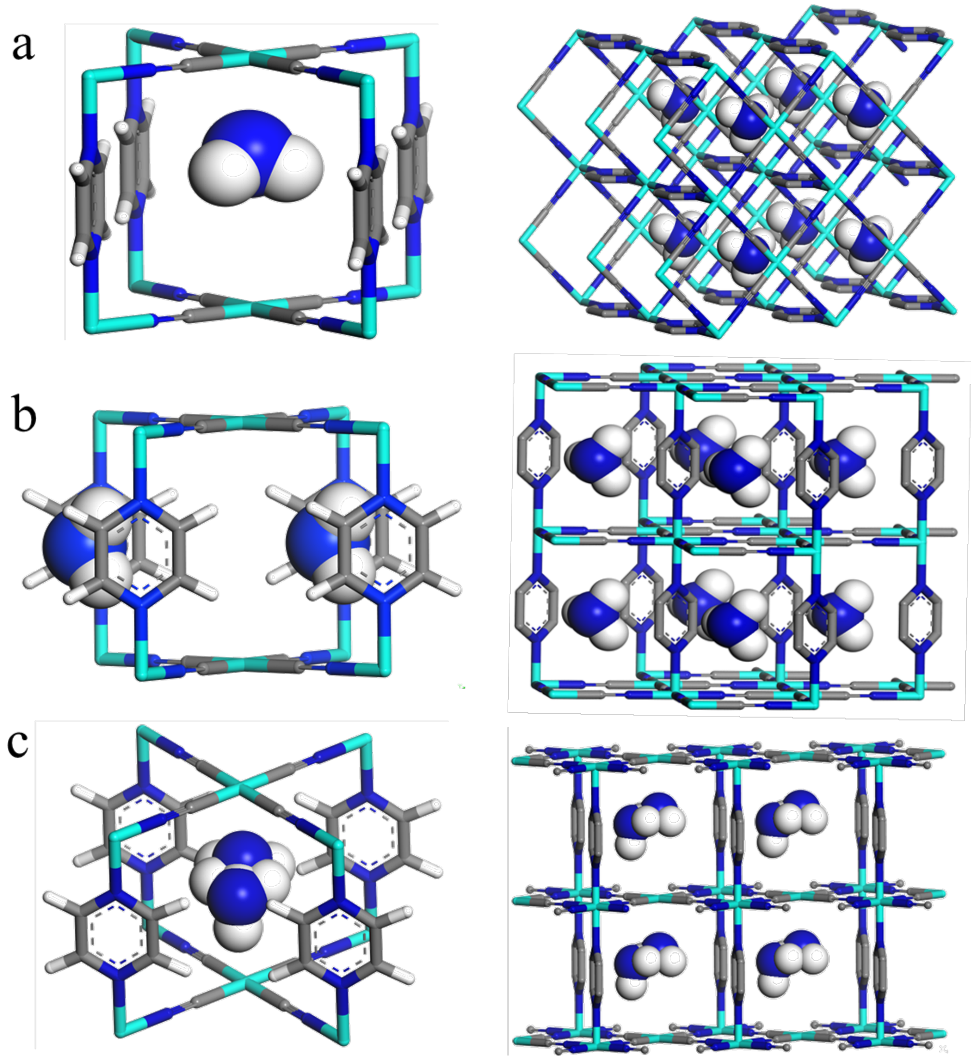

Supplementary Fig. 11. GCMC simulated NH_3 adsorption sites of MNi-Pyz. NH_3 binding sites for (a)site I, (b)site II, (c)site III on NiNi-Pyz.

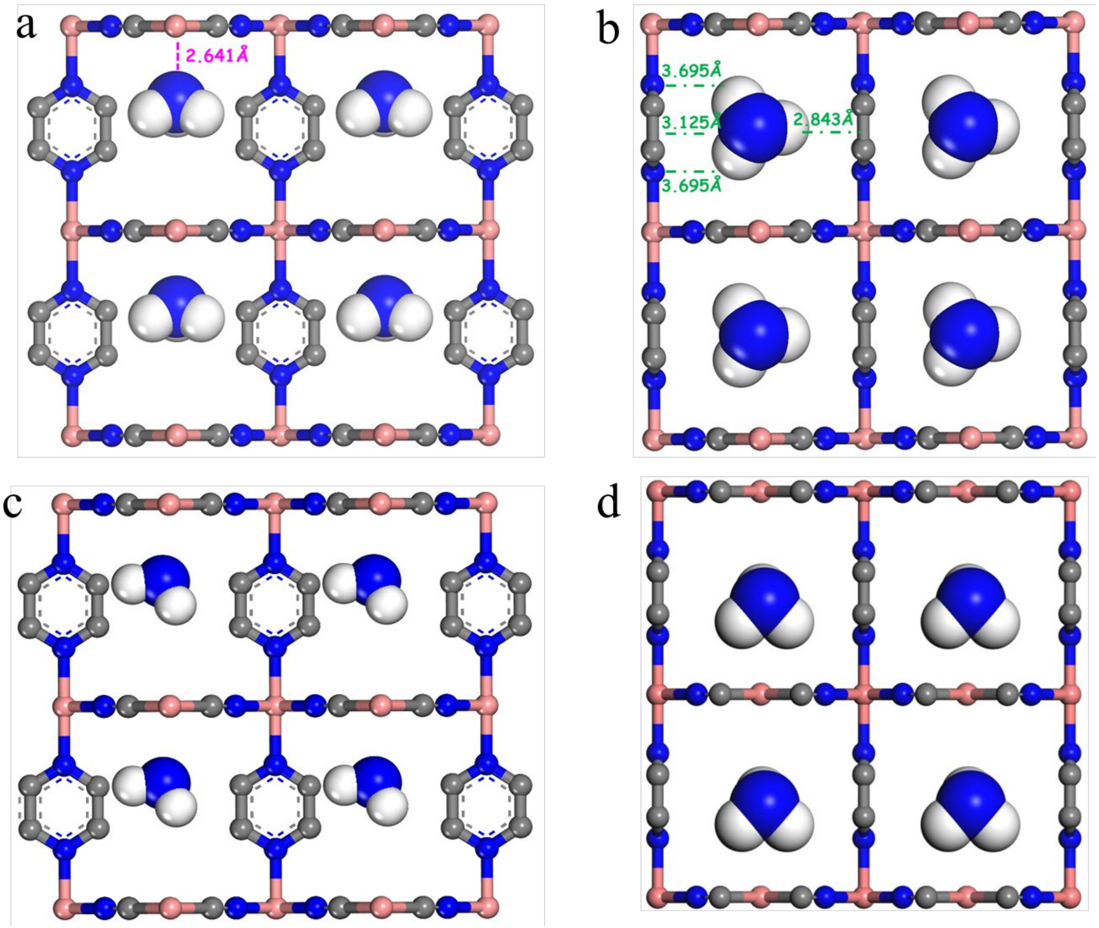

Supplementary Fig. 12. GCMC simulated NH_3 adsorption sites of CoNi-Pyz. NH_3 binding sites for (a) site I, (b) site II, (c) site III and (d) site IV on CoNi-Pyz.

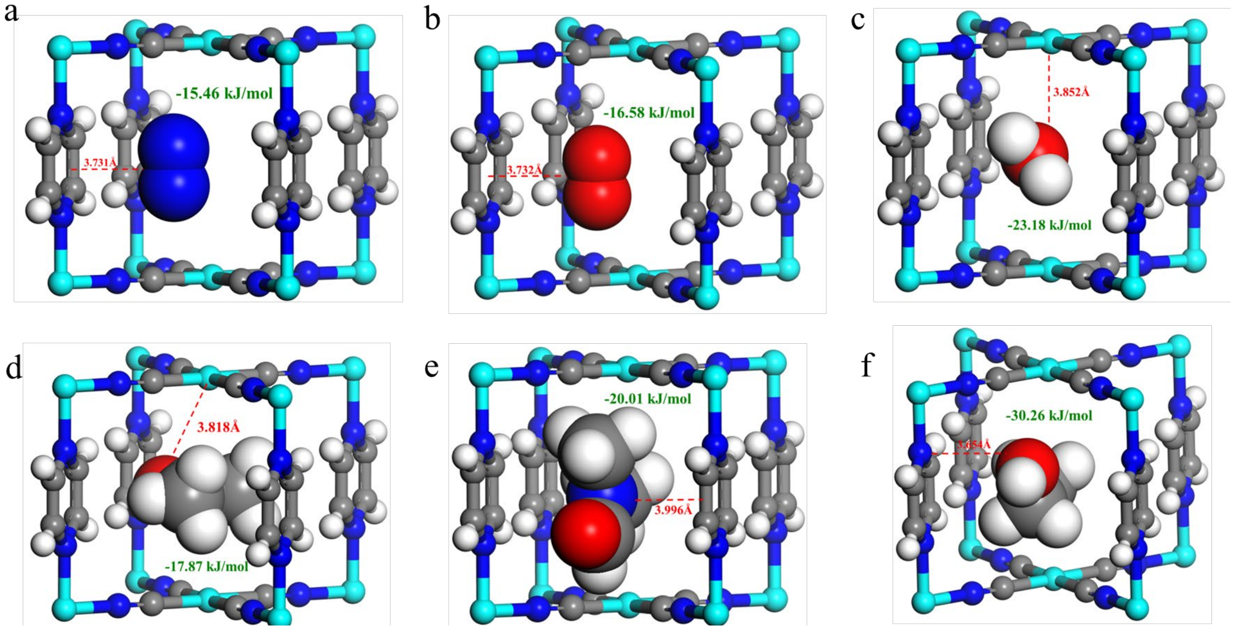

Supplementary Fig. 13. GCMC simulated of NiNi-Pyz. The binding sites and energy of (a) N_2, (b) O_2, (c) H_2O, (d) acetone, (e) DMF, (f) ethanol on NiNi-Pyz, respectively.

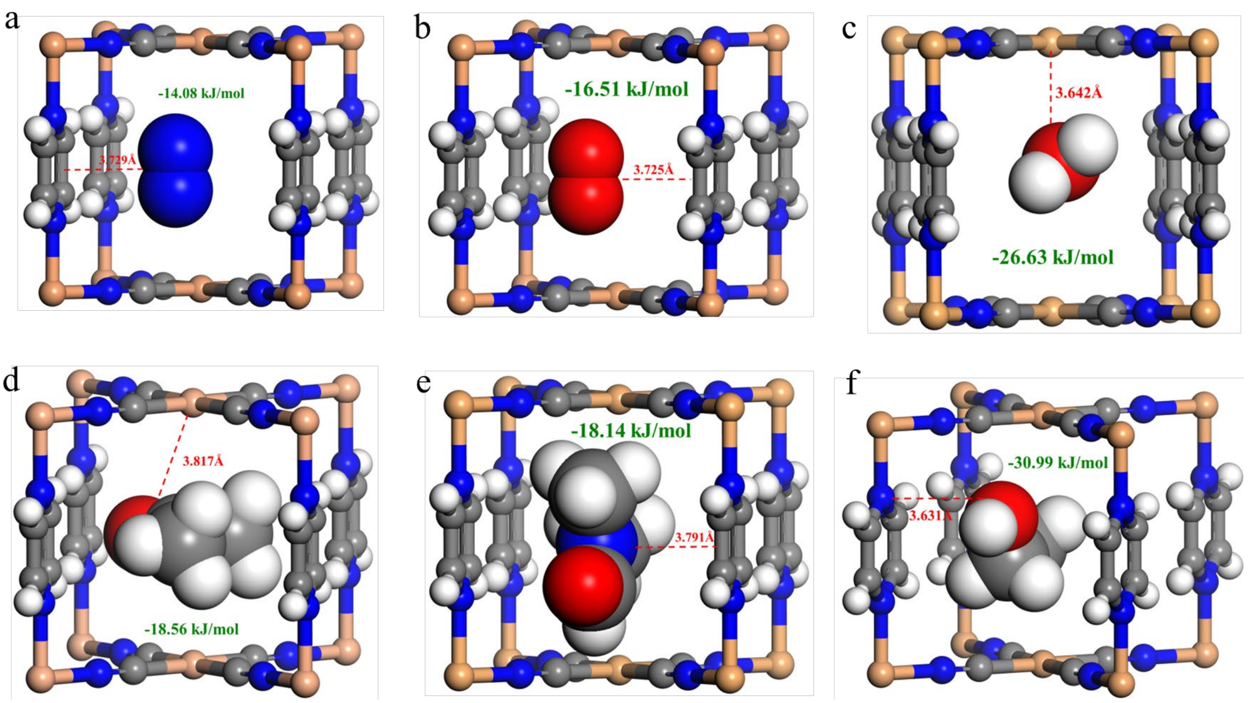

Supplementary Fig. 14. GCMC simulated of CoNi-Pyz. The binding sites and energy of (a) N_2, (b) O_2, (c) H_2O, (d) acetone, (e) DMF, (f) ethanol on CoNi-Pyz, respectively.

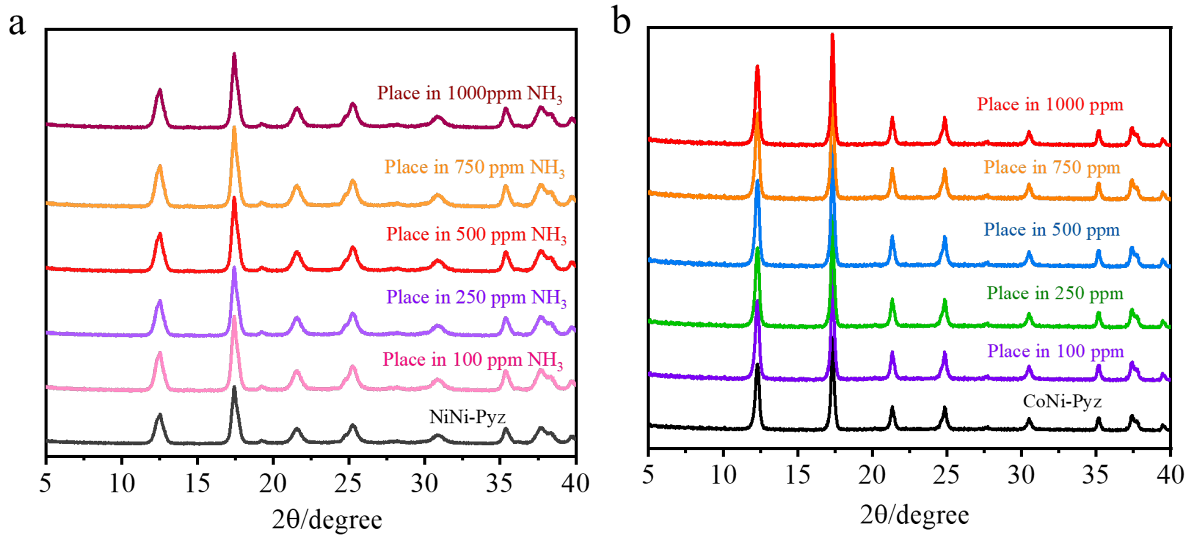

Supplementary Fig. 15. Characterization of MNi-Pyz. PXRD patterns of (a) NiNi-Pyz, (b) CoNi-Pyz after placed at 100 ~ 1000 ppm NH_3 for one day.

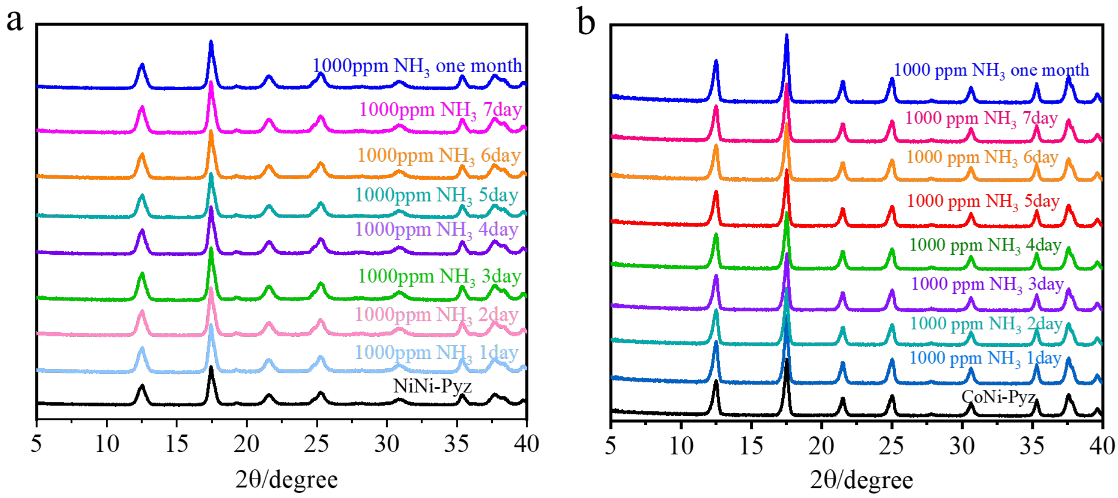

Supplementary Fig. 16. Characterization of MNi-Pyz. PXRD patterns of (a) NiNi-Pyz, (b) CoNi-Pyz after placed at 1000 ppm NH_3 after a week to a month.

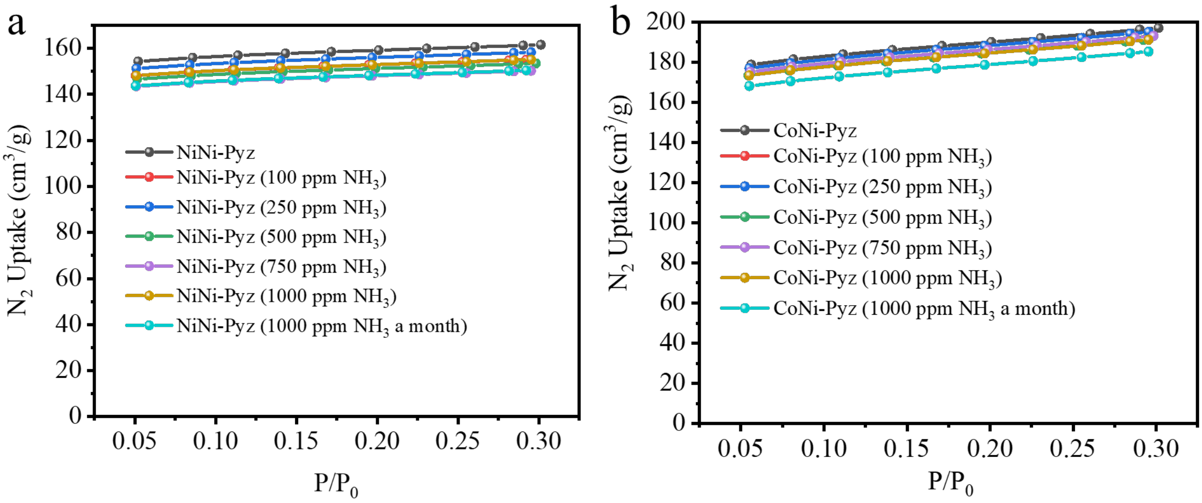

Supplementary Fig. 17. BET characterization of MNi-Pyz. (a) NiNi-Pyz and (b) CoNi-Pyz after NH_3 environment.

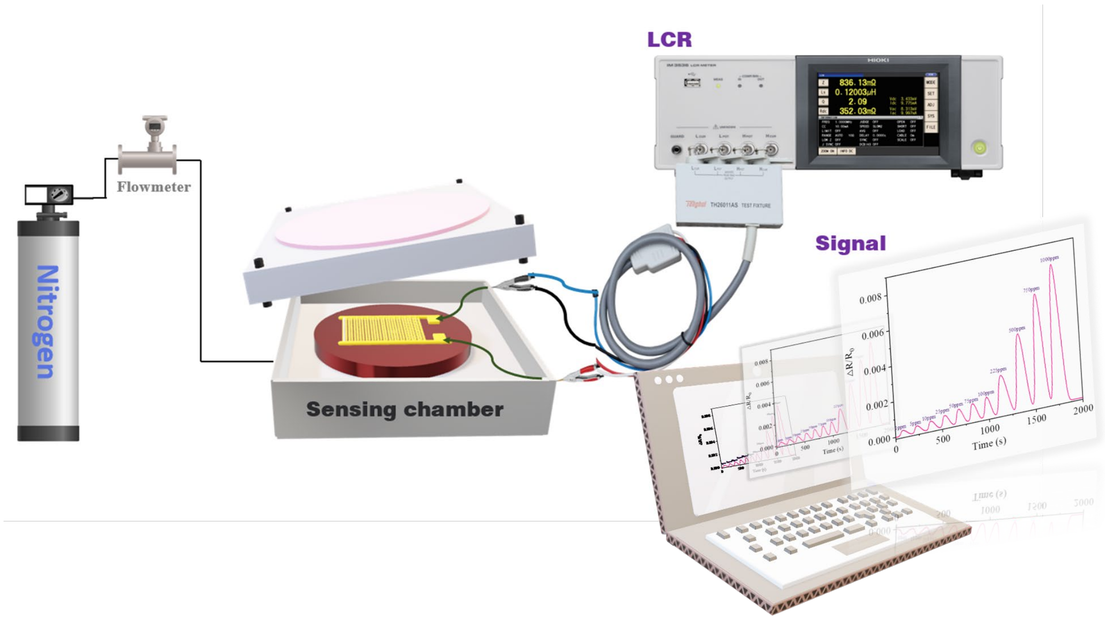

Supplementary Fig. 18. Flow chart of gas sensing device

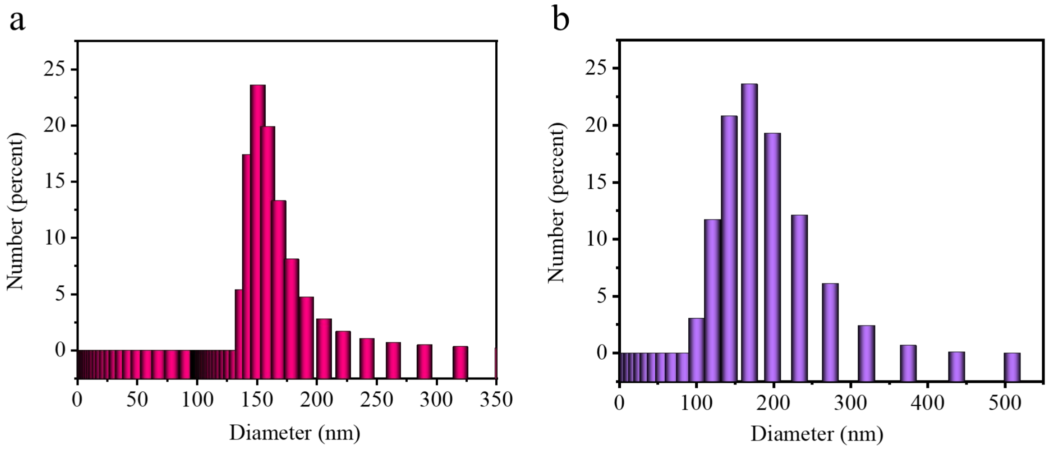

Supplementary Fig. 19. The particle size distribution characterization of MNi-Pyz. (a) NiNi-Pyz and (b) CoNi-Pyz.

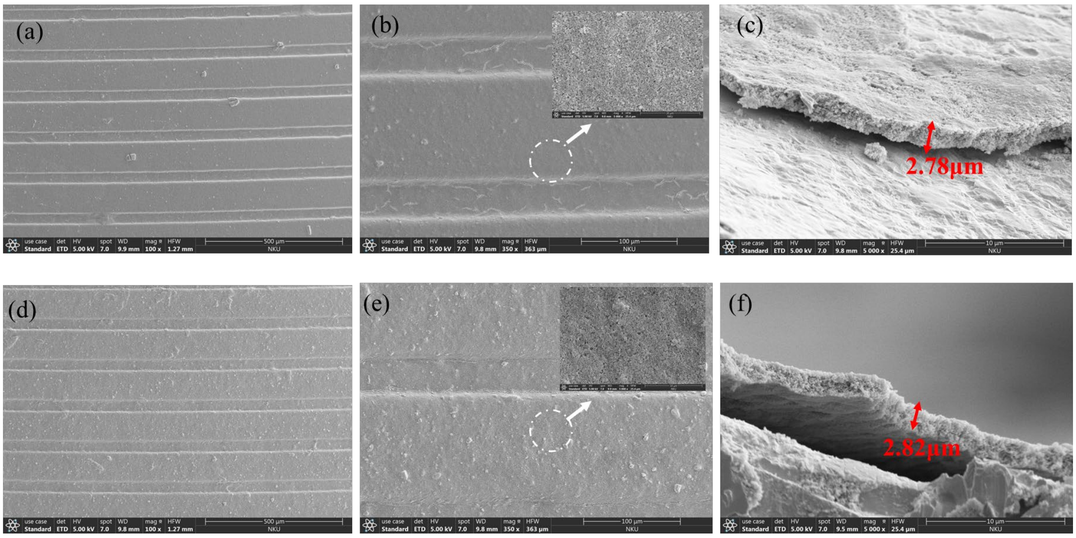

Supplementary Fig. 20. Characterization of MNi-Pyz. FESEM images of (a) NiNi-Pyz and (b) CoNi-Pyz coated on interdigital electrode.

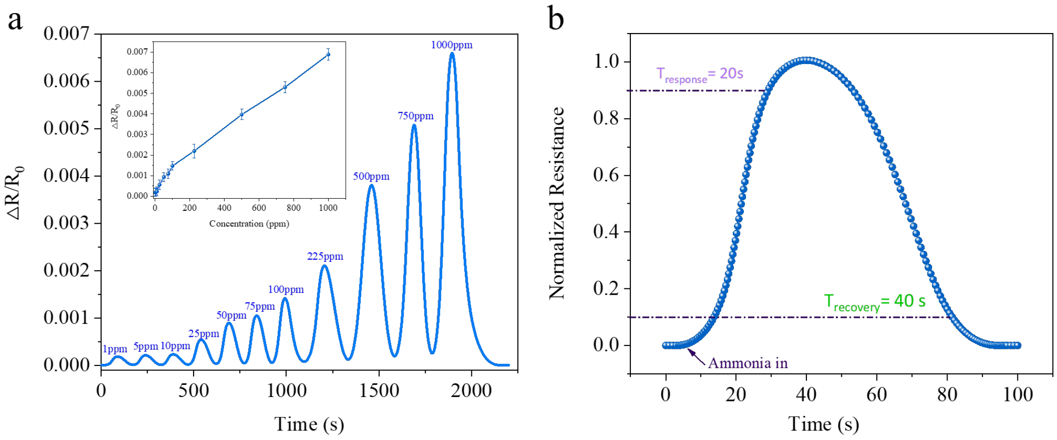

Supplementary Fig. 21. Sensing performance for NH_3. For the CoNi-Pyz (a) Detection of NH_3 in

different ranges of ppm concentrations: from (1~1000 ppm). Insets: Linear response in the corresponding range with error bars depicted in pink. Data show means ± SD (n = 5 replicates). (b) Response–recovery time curves.

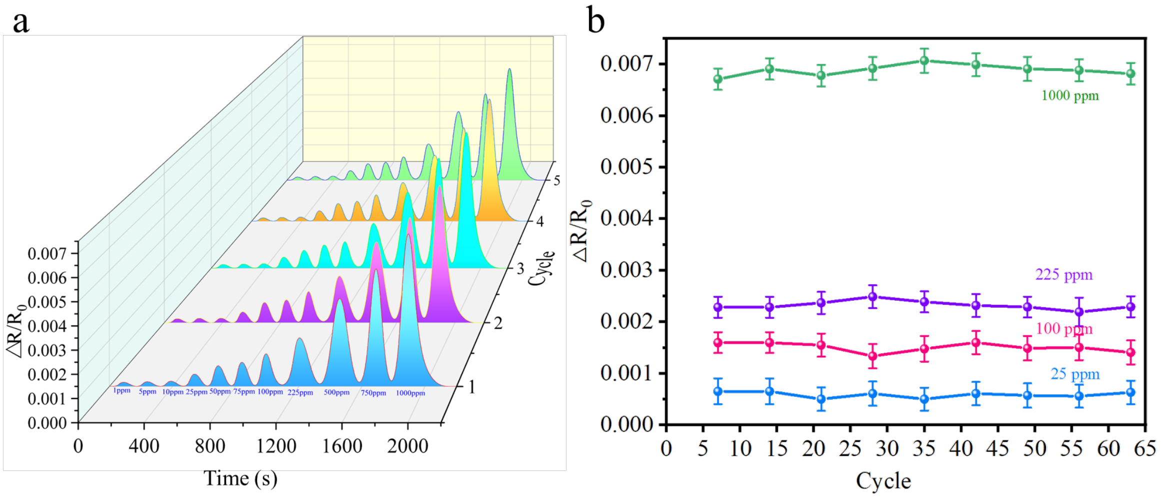

Supplementary Fig. 22. Sensing performance for NH_3. For the CoNi-Pyz (a) Performance of five cycles at 1 ~ 1000 ppm, (b) Stability of ammonia detection for CoNi-Pyz at 20, 115, 225 and 1000 ppm. Data show means ± SD (n = 5 replicates).

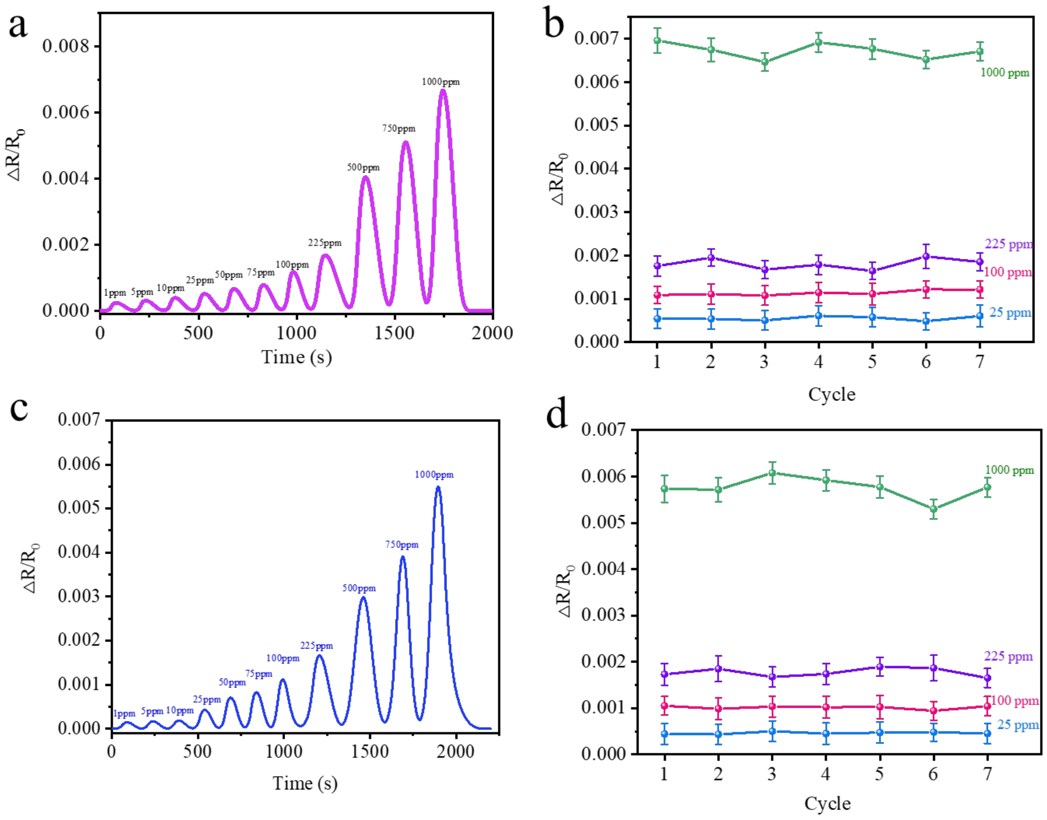

Supplementary Fig. 23. Sensing performance for NH_3. (a, c) Detection of NH_3 in different ranges of ppm concentrations: from (1~1000 ppm). Data show means ± SD (n = 5 replicates). (b, d) Stability

of NH_3 detection at 20, 115, 225 and 1000 ppm. for the NiNi-Pyz and CoNi-Pyz in air environments. Data show means ± SD (n = 5 replicates).

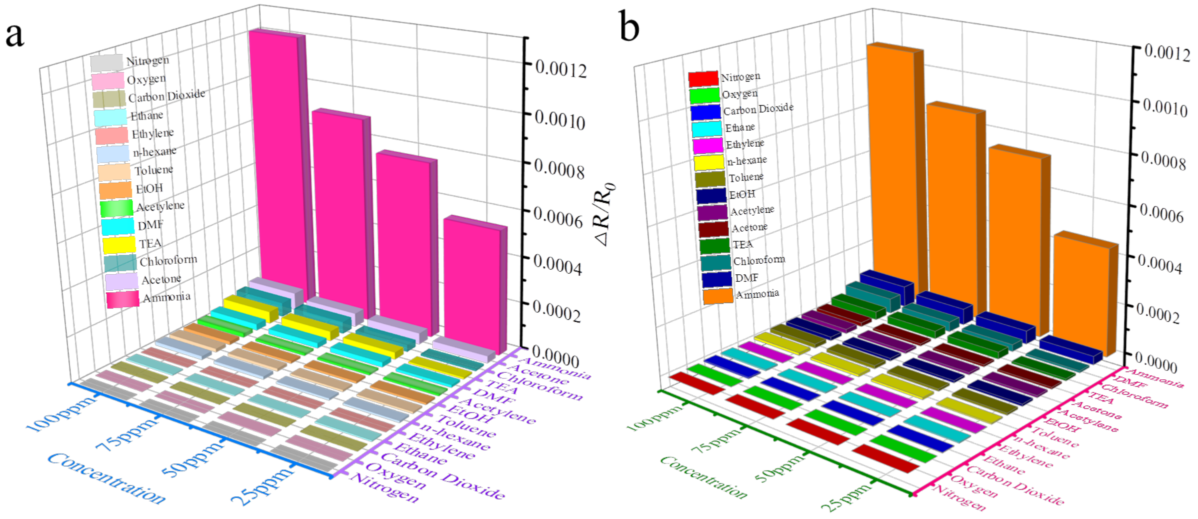

Supplementary Fig. 24. Sensing performance for NH_3. Specific sensing evaluation. Response toward NH_3 and interfering gases for (a) NiNi-Pyz. (b) CoNi-Pyz in air environments.

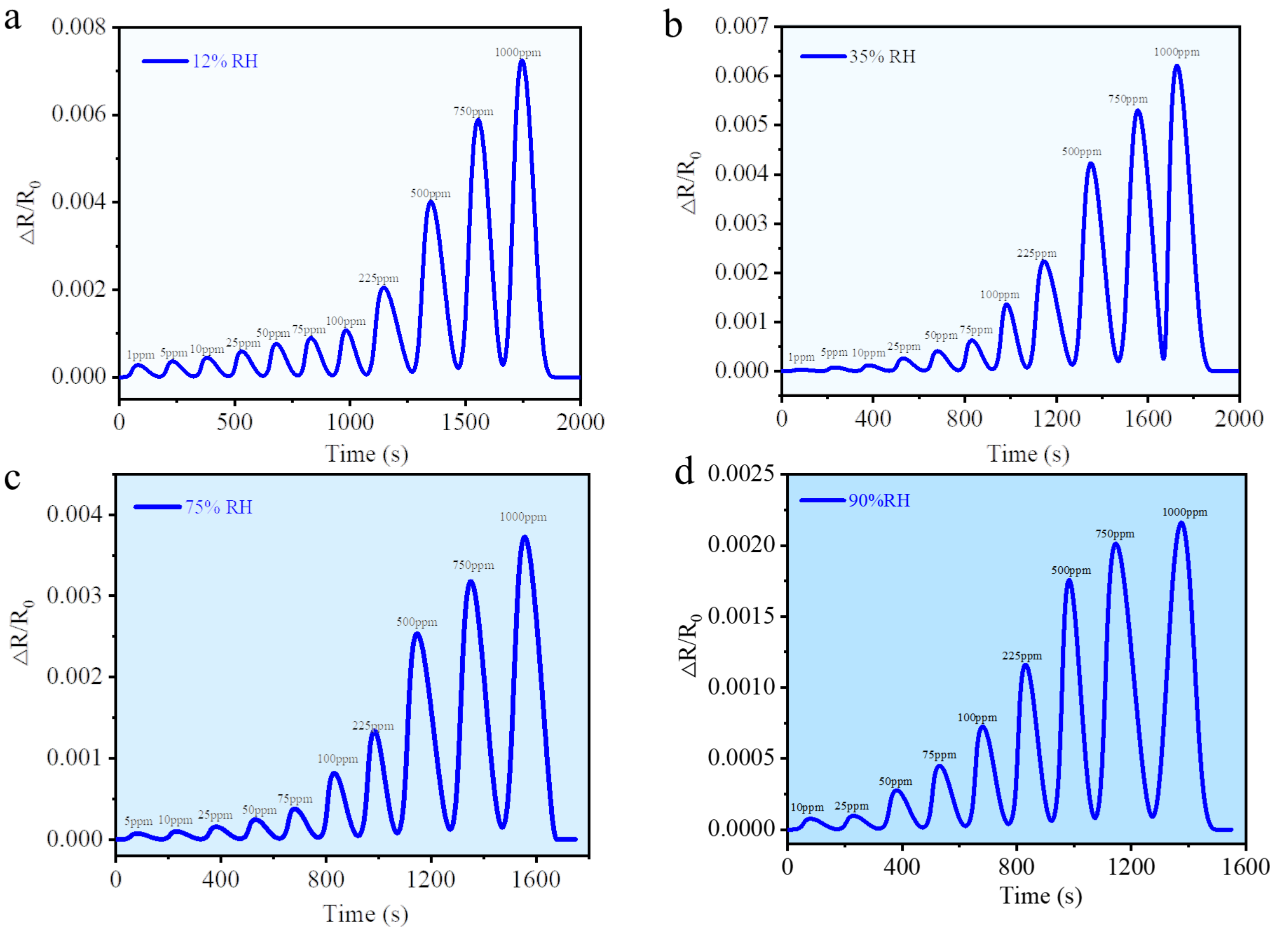

Supplementary Fig. 25. Sensing performance for NH_3. NH_3 sensing performance of NiNi-Pyz exposure to NH_3 at 12%, 35%, 75%, 90% RH.

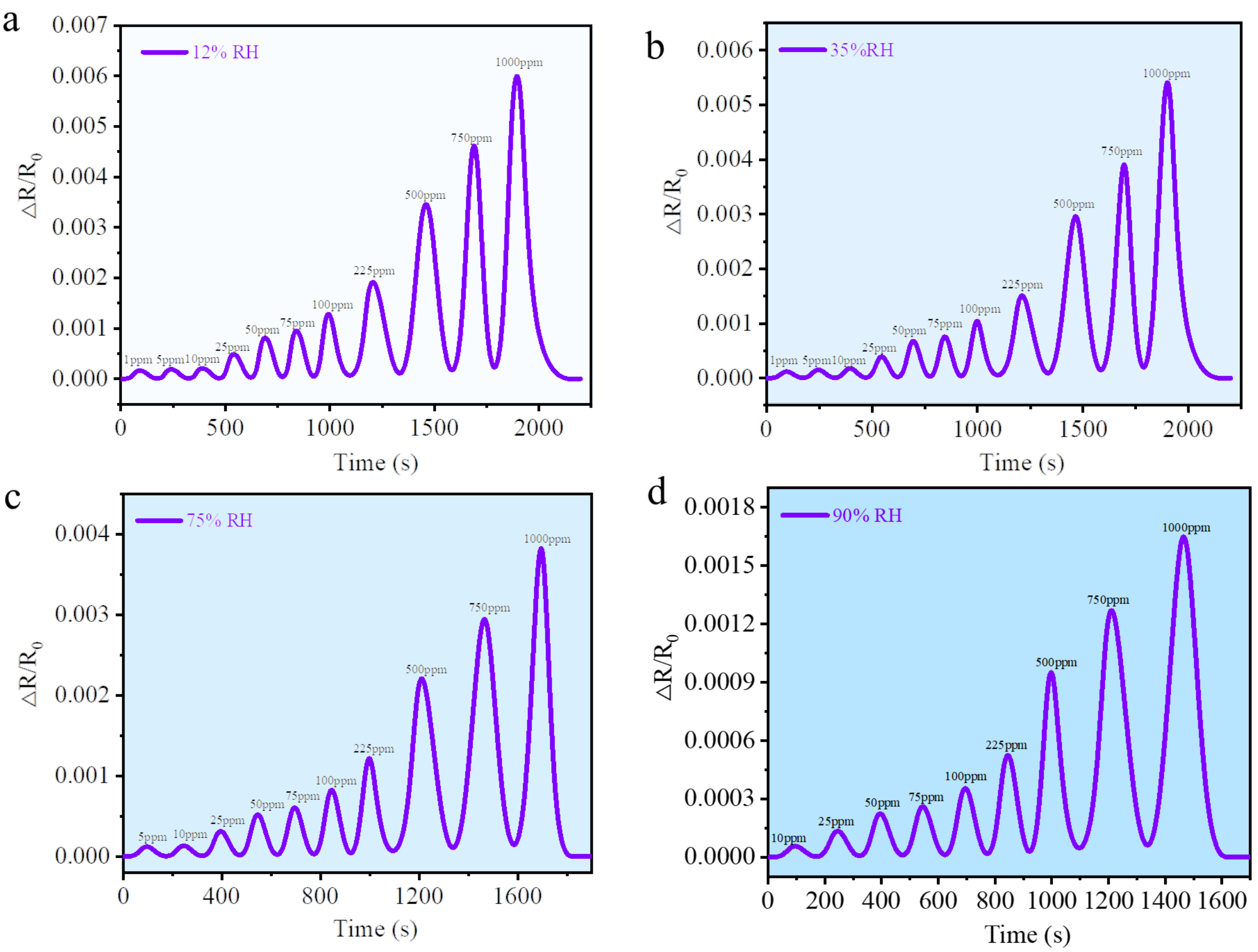

Supplementary Fig. 26. Sensing performance for NH_3. NH_3 sensing performance of CoNi-Pyz exposure to NH_3 at 12%, 35%, 75%, 90% RH.

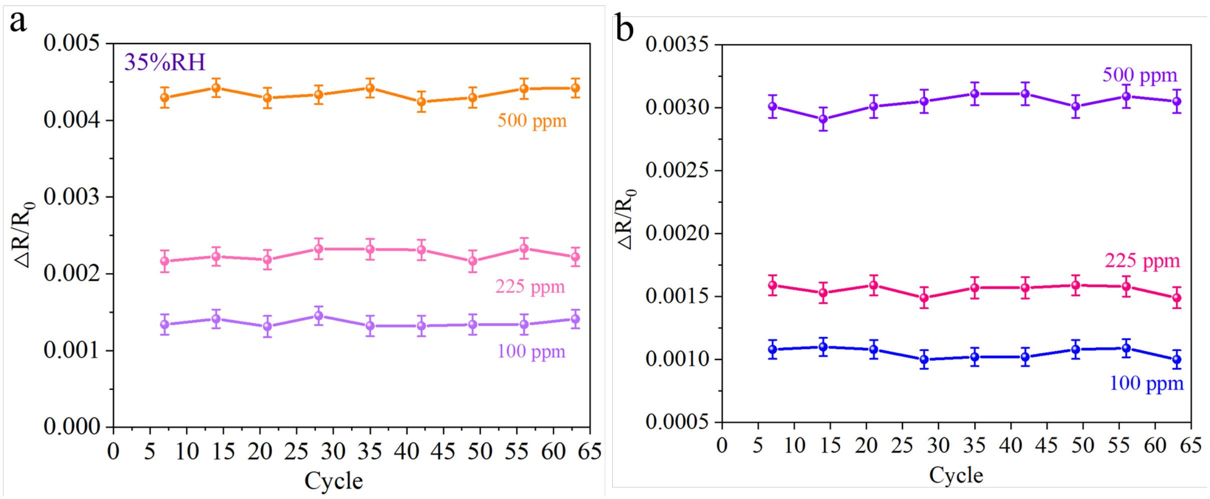

Supplementary Fig. 27. Sensing performance for NH_3. NH_3 sensing performance of (a) NiNi-Pyz, (b) CoNi-Pyz exposure to 100, 225 and 500 ppm of NH_3 for seven cycles at 35% RH for two months. Data show means ± SD (n = 5 replicates).

Supporting Table.

Supplementary Table 1. Compare with other reported ammonia capture materials

Material	Uptake (mmol g^{-1})	Low-pressure uptake (mmol g^{-1} at pressure)	stability	pore size (Å)	Synthetic solvent, Temperature, Time	Regeneration conditions
NiNi-Pyz	21.5	5.5 at 5mbar<br>4 at 0.6 mbar	stable in pH=1 and pH=13 and boiling water	7.2	298K stirring 8min	80 °C 2 h
CoNi-Pyz	29.1	3.1 at 5mbar<br>6.3 at 0.1 bar	stable in pH=1 and pH=13 and boiling water	6.8	298K stirring 8min	80 °C 2 h
LiCl@MIL-53-$(OH)_2$[1]	33.9	11.3 at 0.1bar	TG: 400°C	8	DMF/EtOH/H_2O 125°C 8h then 100 °C 12h, then 120 °C 12h	120°C 8h
Mg_2(dobpdc)[2]	23.9	8.25 at 0.57mbar<br>7.82 at 0.072mbar	NH_4OH vapor for 24h TG: 450°C	18~22	DMF/EtOH microwave reactor 130°C 20min	250 °C 12 h
CoHCC[3]	21.9	8.1 at 1mbar	XRD has one more peak after NH_3 adsorption		Need special grade of the raw materials and micromixer hole diameter	150 °C 24 h
Ni_2(dobpdc)[2]	20.8	5.10 at 0.58 mbar	collapses in NH_4OH vapor TG: 370°C	18~22	DMF/EtOH microwave reactor 110°C 20min	250 °C 12 h
Cu_2Cl_2BBTA[4]	19.7	12 at 0.1bar	Non-crystalline	13	DMF/MeOH 65°C 72h	200°C dynamic vacuum
P1-PO_3H_2[5]	18.7	3.3 at 0.5 mbar	Under low pressure NH_3 reversible, TG curve keeps dropping after 200 °C	11	anhydrous DMF/THF 85°C 24h, hen soak them in ionic solution for 72h	110 °C under vacuum
Fe-MIL-101-SO_3H[6]	18	3.52 at 0.510 mbar	Irreversible, TG: 400°C	-	H_2O 180°C 72h	125 °C 24 h
Co_2Cl_2BBTA[4]	17.9	5 at 0.75mbar<br>11.8 at 0.1bar	collapses	13	DMF/MeOH 65°C 72h	200 °C dynamic vacuum
3D-[$Zn_2(L1)_2$(bpe)][7]	17.8[a]	8 at 0.1 bar	collapses	8 Å	DEF 80°C 1h	40 °C 12 h under vacuum.
Cu(cyhdc)[8]	17.5	16 at 80 mbar	large volume expansion upon NH_3 adsorption	NA	NA	175 °C for 3 h
$Co(NA)_2$[9]	17.5	3 at 0.1 bar	Order disorder transition TG 300°C	3.8 Å,	2D DMF 80°C 4h+180°C 1h	150 °C 70 min
MFM-300(V^{IV})[10]	17.3[a]	7 at 0.1bar[a]	Hysteresis loop	5.4	H_2O 210°C 72h	373 K under vacuum.
OH-DUT-6[11]	16.4	4.7 at 1.01mbar	Not completely reversible	23、16、13	DMF/EtOH 85°C 10h	Supercritical CO_2 for 5h
MFM-300(V^{III})[10]	15.6[a]	4.1 at 0.1bar[a]	Residual NH_3 content is ~9%, after NH_3	5.2	H_2O 210°C 72h	200 °C for 24 h.
Mn_2Cl_2(BTDD)$(H_2O)_2$[12]	15.4	6 at 0.1bar	BET decrease TG:450°C	21.4	DMF/EtOH 65°C 240h	200°C dynamic vacuum
PCN-250[13]	14.7	6 at 0.1bar<br>3.5 at 0.005 bar	a loss in uptake ~11.4 mmol g^{-1}. TG 400°C	6 and 9.3	H_2O/HAc 95°C 24h	250 °C 6 h
Ni_2Cl_2BBTA[4]	14.6	10 at 0.1bar	retains its crystallinity and porosity	13	DMF/MeOH 65°C 72h	200 °C dynamic

Material	Uptake (mmol g^{-1})	Low-pressure uptake (mmol g^{-1} at pressure)	stability	pore size (Å)	Synthetic solvent, Temperature, Time	Regeneration conditions
MFM-300(Cr)[10]	14[a]	4.8 at 0.1bar [a]	Residual NH_3 content is ~9%	-	H_2O 210°C 72h	vacuum 200 °C for 24 h.
MFM-300(Al)[14]	13.9	6.3 at 0.1bar	Residual NH_3 content is ~9%	7.5	H_2O 210°C 72h	200 °C for 24 h.
MFM-300(Sc)[15]	13.1	4 at 0.1bar	Residual NH_3 content is ~9%	8.1	THF/DMF 75°C 72h	-
MOF-5[16]	12.2	6 at 0.1 bar	collapses in NH_3	9	Vacuum DEF 90°C 24h	Drying at 70 ° C for 3 days, stored in chloroform
Ni_2Cl_2(BTDD)(H_2O)$_2$[12]	12	5.8 at 0.1bar	TG curve continuous decline after 200°C	21.4	DMF/EtOH 65°C 240h	200 °C dynamic vacuum
HKUST-1[17]	12.1 [b]	5.5 at 0.1 bar	collapses in NH_3	6~10	DMF/H_2O 85°C 21h	120 °C for 6 h
Co_2Cl_2(BTDD)(H_2O)$_2$[12]	12	3.5 at 4mbar	TG curve continuous decline after 200°C	21.4	DMF/EtOH 65°C 240h	200 °C dynamic vacuum
UiO-66-NH_3Cl[6]	12	2.64 at 0.663 mbar	Irreversible TG: 250°C	-	DMF 120°C 24h	Difficult reactivation
Amberlyst 15[18]	11.34	0.376 at 0.071 mbar 1.114 at 0.113 mbar	Commercial materials	-	Rohm & Haas Co	115 °C 1h
Ga-PMOF[19]	10.5	1.5 at 0.05bar	Adsorption reduced, not desorption TG 350°C	10	DMF 120°C 15h	140 °C 12 h
PFC-27/CF_3SO_3[20]	10.4	8.71 at 0.1bar	50%RH, the capacity drops sharply. TG 260°C	12~20	H_2O/MeOH 120°C 48h	120 °C 12 h under vacuum
UiO-66-NH_2[6]	10.3	0.93 at 0.488mbar	Irreversible TG 350°C	8~11	DMF 120°C 24h	100 °C 24 h
Zn(NA)$_2$[9]	10.2	4 at 0.3 bar	becomes rougher after NH_3, easily expanded. TG: 300°C	3.8	2D DMF 180°C 4h	150 °C 70 min
MIL-101[21]	10	2.8 at 0.1 bar	TG: 300°C	29	TMAOH 180°C 24h	25 °C 30 min
In-PMOF[19]	9.4	3.5 at 0.05bar	Adsorption reduced, not desorption TG 350°C	10	DMF 120°C 15h	140 °C 12 h
13X Zeolite[18] (Lancaster 6149)	9.3	1.264 at 0.01 mbar 3.786 at 0.58 mbar	Commercial materials	0.4~0.8 mm	Lancaster Synthesis	300 °C 1 h
PI[22]	9	0.4 at 1 mbar	23% loss	~9 Å	m-cresol 200°C 6day	250 °C 1 h
1TCS[23]	8.5	0.92 at 0.52 mbar	10 cycles TG 200°C		1TC+H_2O+Chlorosulfonic acid 5.5days	120 °C 10 h
NU-300[24]	8.2	1.5 at 0.01bar	Adsorption loss TG 400°C pH=2 and boiled water for one day, pH=11, half collapsed	11	DMF 120°C 3day	120 °C overnight
MIL-53[21]	4.4	0.5 at 0.1 bar	TG 400°C	12~17	H2O 220°C 3s	25 °C 30 min
1T[23]	3.8	0.04 at 0.49 mbar	TG 500°C	-	FDA/toluene/ DCE microwave 5h	120 °C 10 h

[a] (273 K); [b] (301 K 1.2 bar)

Supplementary Table 2. Adsorption energies between NH_3 and MNi-Pyz

Material	Adsorption Energy (kJ mol^{-1})			
	Site 1	Site 2	Site 3	Site 4
**NiNi-Pyz**	-41.68	-34.23	-27.60	--
**CoNi-Pyz**	-40.56	-37.02	-31.30	-23.61

Supplementary Table 3. LOD for materials at different humidity.

Material	LOD (ppm)				
	Pure N_2	12% RH	35% RH	75% RH	90% RH
**NiNi-Pyz**	0.025	0.028	0.035	0.051	0.088
**CoNi-Pyz**	0.097	0.103	0.110	0.139	0.178

Supplementary Table 4[a]. Comparison of room-temperature NH_3 sensing of MOFs.

Material	Detection range(ppm)	Response time （min）	Recovery time （min）	LOD （ppm）
**NiNi-Pyz**	**1-1000**	**0.08**	**0.92**	**0.025**
**CoNi-Pyz**	**1-1000**	**0.33**	**0.67**	**0.097**
Cu-HHTP 3D thin film[25]	0.005–100	0.58	15	8.7×10^{-5}
Cu-BHT film[26]	1-100	0.97	1.7	0.23
Cu-HHTP 2D thin film[27]	1–100	1.36	9.11	0.5
Cu_3(HHTP)-THQ[28]	1–100	1.65	2.57	0.35
Cu_3HITP_2[29]	0.5-10	1.7	3.34	0.5
Cu-HHTP nanorods thick film[30]	2.5-80	3	10	NA
RE-fcu-MOF[31]	1-100	4.17	NA	0.092
NiPc-Ni[32]	2-80	30	incomplete	0.31
NiPc-Cu[32]	2-80	30	30	0.33
CuPc@IRMOF-3[33]	0.5–50	NA	NA	0.052
Cu-HHTP-Cu-TCPP[34]	1-100	NA	NA	NA
PANI/UiO-0.13[35]	1-100	0.42	0.23	NA
SiO_2CuOF-graphene-PAni[36]	1–100	0.67	3.34	0.6
Pd-Co@IRMOF-1[37]	1-90	0.77	0.37	1
MoS2/FDM-23[38]	0.1-100	1.33	2.4	0.1
M_3HHTP_2/Graphene[39]	5–1200	5	10	5
Cu-BTC@GO[40]	100–500	20	30	100

[a]: the upper part of the Table S4 is pure MOFs, and the lower part is MOF-doped polymer materials.

Supplementary Table 5. Comparison of room-temperature NH_3 sensing electrical signal materials.

Material	Detection range(ppm)	Response time (min)	Recovery time (min)	LOD (ppm)
**NiNi-Pyz**	**1-1000**	**0.08**	**0.92**	**0.025**
**CoNi-Pyz**	**1-1000**	**0.33**	**0.67**	**0.75**
pDPPBu-BT[41]	0.01–1000	0.083	0.2	0.01
Polypyrrole nanotubes[42]	0.00005-2	0.17	0.34	5×10^{-5}
rGo–WO_3 nanocomposites[43]	10–100	0.28	0.4	1.14
Ni-doped ZnO thin films[44]	25–1000	0.77	0.23	25
rGO/ZnO nanowire[45]	0.5–5000	0.83	3.33	0.5
Core/Shell CeO_2-PANI Nanoparticles[46]	2–100	0.96	6	2
MoS_2/ZnO nanocomposite films[47]	0.25–100	0.1	0.18	0.0012
Coral-shaped Dy_2O_3[48]	0.1-100	0.1	14	0.1
PANI/MWCNTs[49]	2–10	0.1	0.58	2
Ag/rGO[50]	1500-10000	0.1	0.17	NA
SnO_2-rGO[51]	20–500	0.13	0.22	20
PEDOT[52]	10-100	0.17	1	10
n-MoS_2/p-CuO[53]	5–500	0.28	0.43	5
MnO_2 nanofibers[54]	1–100	1.17	1.45	1
PPTA-NiO@CuO[55]	20–100	1.21	0.58	4.65×10^{-6}
MoS_2/Co_3O_4[56]	0.1–5	1.63	2.27	0.1
MoS_2 thin fIlms[57]	0.3–30	2		0.3
WS_2[58]	1–5	2.5	4.8	1.2
WS_2/TiO_2 QD[59]	20–500	3.33	3	20
SWCNT[60]	0.02–3	3.34	11.2	0.003
CuTCNQ[61]	10-99	4	14	10
Modified SWCNT[62]	1.5–20	5	15	0.1
2D $Ti_3C_2T_x$[63]	0.1–100	5	10	0.13
SnO_2/rGO[64]	20-1000	13.5	14	NA
Graphene nanomesh[65]	5–100	15	20	0.16
Activated carbons[66]	10–500	30	10	10
PbS_2 QDs/TiO_2[67]	2–100	-	-	2

Supplementary Table 6. Antoine constants.

Gas	A	B	C
n-hexane	6.87776	1171.53	224.366
Toluene	6.95464	1344.8	219.48
EtOH	8.32109	1718.1	237.52
Acetone	7.11714	1210.595	229.664
TEA	6.8264	1161.4	205
Chloroform	6.4934	929.44	196.03
DMF	7.3438	1624.7	216.2

Supplementary references

1 Shi, Y., Wang, Z., Li, Z., Wang, H., Xiong, D., Qiu, J., ... & Wang, J. Anchoring LiCl in the Nanopores of Metal–Organic Frameworks for Ultra-High Uptake and Selective Separation of Ammonia. *Angew. Chem. Int. Ed.* **61**, e202212032 (2022).

2 Kim, D. W., Kang, D. W., Kang, M., Lee, J. H., Choe, J. H., Chae, Y. S., ... & Hong, C. S. High Ammonia Uptake of a Metal-Organic Framework Adsorbent in a Wide Pressure Range. *Angew. Chem. Int. Ed.*, **59**, 22531-22536 (2020).

3 Takahashi, A., Tanaka, H., Parajuli, D., Nakamura, T., Minami, K., Sugiyama, Y., ... & Kawamoto, T. Historical Pigment Exhibiting Ammonia Gas Capture beyond Standard Adsorbents with Adsorption Sites of Two Kinds. *J. Am. Chem. Soc.*, **138**, 6376-6379 (2016).

4 Rieth, A. J., & Dincă, M. Controlled Gas Uptake in Metal-Organic Frameworks with Record Ammonia Sorption. *J. Am. Chem. Soc.* **140**, 3461-3466 (2018).

5 Barin, G., Peterson, G. W., Crocella, V., Xu, J., Colwell, K. A., Nandy, A., ... & Long, J. R. Highly effective ammonia removal in a series of Brønsted acidic porous polymers: investigation of chemical and structural variations. *Chem. Sci.* **8**, 4399-4409 (2017).

6 Van Humbeck, J. F., McDonald, T. M., Jing, X., Wiers, B. M., Zhu, G., & Long, J. R. Ammonia capture in porous organic polymers densely functionalized with Brønsted acid groups. *J. Am. Chem. Soc.* **136**, 2432-2440 (2014).

7 Glomb, S., Woschko, D., Makhloufi, G., & Janiak, C. Metal–Organic Frameworks with Internal Urea-Functionalized Dicarboxylate Linkers for SO_2 and NH_3 Adsorption. *ACS Appl. Mater. Interfaces*. **9**, 37419-37434 (2017).

8 Snyder, B. E., Turkiewicz, A. B., Furukawa, H., Paley, M. V., Velasquez, E. O., Dods, M. N., & Long, J. R. A ligand insertion mechanism for cooperative NH_3 capture in metal–organic frameworks. *Nature*. **613**, 287-291 (2023).

9 Chen, Y., Shan, B., Yang, C., Yang, J., Li, J., & Mu, B. Environmentally friendly synthesis of flexible MOFs $M(NA)_2$ (M = Zn, Co, Cu, Cd) with large and regenerable ammonia capacity. *J. Mater. Chem. A*. **6**, 9922-9929 (2018).

10 Han, X., Lu, W., Chen, Y., Da Silva, I., Li, J., Lin, L., ... & Schroder, M. High Ammonia Adsorption in MFM-300 Materials: Dynamics and Charge Transfer in Host-Guest Binding. *J. Am. Chem. Soc.* **143**, 3153-3161 (2021).

11 Spanopoulos, I., Xydias, P., Malliakas, C. D., & Trikalitis, P. N. A Straight Forward Route for the Development of Metal–Organic Frameworks Functionalized with Aromatic −OH Groups: Synthesis, Characterization, and Gas (N_2, Ar, H_2, CO_2, CH_4, NH_3) Sorption Properties. *Inorg. Chem.* **52**, 855-862 (2013).

12 Rieth, A. J., Tulchinsky, Y., & Dinca, M. High and Reversible Ammonia Uptake in Mesoporous Azolate Metal-Organic Frameworks with Open Mn, Co, and Ni Sites. *J. Am. Chem. Soc. 138*, 9401-9404 (2016).

13 Chen, Z., Wang, X., Cao, R., Idrees, K. B., Liu, X., Wasson, M. C., & Farha, O. K. Water-Based Synthesis of a Stable Iron-Based Metal–Organic Framework for Capturing Toxic Gases. *ACS Mater. Lett.* **2**, 1129-1134 (2020).

14 Godfrey, H. G., da Silva, I., Briggs, L., Carter, J. H., Morris, C. G., Savage, M., ... & Schröder, M. Ammonia Storage by Reversible Host–Guest Site Exchange in a Robust Metal–Organic Framework. *Angew. Chem. Int. Ed.* **130**, 14994-14997 (2018).

15 Lyu, P., Wright, A. M., López-Olvera, A., Mileo, P. G., Zárate, J. A., Martínez-Ahumada, E., ... & Maurin, G. Ammonia Capture via an Unconventional Reversible Guest-Induced Metal-Linker Bond Dynamics in a Highly Stable Metal–Organic Framework. *Chem. Mater.* **33**, 6186-6192 (2021).

16 Saha, D., & Deng, S. Ammonia adsorption and its effects on framework stability of MOF-5 and MOF-177. *J. Colloid Interface Sci.* **348**, 615-620 (2010).

17 Petit, C., Huang, L., Jagiello, J., Kenvin, J., Gubbins, K. E., & Bandosz, T. J. Toward Understanding Reactive Adsorption of Ammonia on Cu-MOF/Graphite Oxide Nanocomposites. *Langmuir*, **2011**, *27*, 13043-13051.

18 Helminen, J., Helenius, J., Paatero, E., & Turunen, I. Adsorption Equilibria of Ammonia Gas on Inorganic and Organic Sorbents at 298.15 K. *J. Chem. Eng. Data*. **46**, 391-399 (2001).

19 Moribe, S., Chen, Z., Alayoglu, S., Syed, Z. H., Islamoglu, T., & Farha, O. K. Ammonia Capture within Isoreticular Metal–Organic Frameworks with Rod Secondary Building Units. *ACS Mater. Lett.* **1**, 476-480 (2019).

20 Khanpour Matikolaei, M., & Binaeian, E. Boosting Ammonia Uptake within Metal–Organic Frameworks by Anion Modulating Strategy. *ACS Appl. Mater. Interfaces*. **13**, 27159-27168 (2021).

21 Chen, Y., Zhang, F., Wang, Y., Yang, C., Yang, J., & Li, J. Recyclable ammonia uptake of a MIL series of metal-organic frameworks with high structural stability. *Microporous Mesoporous Mater.*, **258**, 170-177 (2018).

22 Lee, J. W., Barin, G., Peterson, G. W., Xu, J., Colwell, K. A., & Long, J. R. A Microporous Amic Acid Polymer for Enhanced Ammonia Capture. *ACS Appl. Mater. Interfaces*. **9**, 33504-33510 (2017).

23 Kang, D. W., Kang, M., Moon, M., Kim, H., Eom, S., Choe, J. H., ... & Hong, C. S. PDMS-coated hypercrosslinked porous organic polymers modified via double postsynthetic acidifications for ammonia capture. *Chem. Sci.* **9**, 6871-6877 (2018).

24 Chen, Y., Zhang, X., Ma, K., Chen, Z., Wang, X., Knapp, J., ... & Farha, O. K. Zirconium-Based Metal–Organic Framework with 9-Connected Nodes for Ammonia Capture. *ACS Appl. Nano Mater.* **2**, 6098-6102 (2019).

25 Lin, Y., Li, W. H., Wen, Y., Wang, G. E., Ye, X. L., & Xu, G. Layer-by-Layer Growth of Preferred-Oriented MOF Thin Film on Nanowire Array for High-Performance Chemiresistive Sensing. *Angew. Chem. Int. Ed.* **60**, 25758-25761 (2021).

26 Chen, X., Lu, Y., Dong, J., Ma, L., Yi, Z., Wang, Y., ... & Liu, Y. Ultrafast In Situ Synthesis of Large-Area Conductive Metal–Organic Frameworks on Substrates for Flexible Chemiresistive Sensing. *ACS Appl. Mater. Interfaces*. **12**, 57235-57244 (2020).

27 Yao, M. S., Lv, X. J., Fu, Z. H., Li, W. H., Deng, W. H., Wu, G. D., & Xu, G. Layer-by-Layer Assembled Conductive Metal–Organic Framework Nanofilms for Room-Temperature Chemiresistive. *Angew. Chem. Int. Ed.*, **56**, 16510-16514 (2017).

28 Yao, M. S., Zheng, J. J., Wu, A. Q., Xu, G., Nagarkar, S. S., Zhang, G., ... & Kitagawa, S. A Dual-Ligand Porous Coordination Polymer Chemiresistor with Modulated Conductivity and Porosity. *Angew. Chem. Int. Ed.*, **59**, 172-176 (2020).

29 Campbell, M. G., Sheberla, D., Liu, S. F., Swager, T. M., & Dincă, M. Cu_3(hexaiminotriphenylene)$_2$: an electrically conductive 2D metal-organic framework for chemiresistive sensing. *Angew. Chem. Int. Ed.* **54**, 4349-4352 (2015).

30 Smith, M. K., Jensen, K. E., Pivak, P. A., & Mirica, K. A. Direct Self-Assembly of Conductive Nanorods of Metal–Organic Frameworks into Chemiresistive Devices on Shrinkable Polymer Films. *Chem. Mater.* **28**, 5264-5271 (2016).

31 Assen, A. H., Yassine, O., Shekhah, O., Eddaoudi, M., & Salama, K. N. MOFs for the Sensitive Detection of Ammonia: Deployment of fcu-MOF Thin Films as Effective Chemical Capacitive Sensors. *ACS Sens.* **2**, 1294-1301 (2017).

32 Meng, Z., Aykanat, A., & Mirica, K. A. Welding Metallophthalocyanines into Bimetallic Molecular Meshes for Ultrasensitive, Low-Power Chemiresistive Detection of Gases. *J. Am. Chem. Soc.* **141**, 2046-2053 (2019).

33 Zheng, J., Pang, K., Liu, X., Li, S., Song, R., Liu, Y., & Tang, Z. Integration and Synergy of Organic Single Crystals and Metal–Organic Frameworks in Core–Shell Heterostructures Enables Outstanding Gas Selectivity for Detection. *Adv. Funct. Mater.* **30**, 2005727 (2020).

34 Yao, M. S., Xiu, J. W., Huang, Q. Q., Li, W. H., Wu, W. W., Wu, A. Q., ... & Xu, G. Van der Waals Heterostructured MOF-on-MOF Thin Films: Cascading Functionality to Realize Advanced Chemiresistive Sensing. *Angew. Chem. Int. Ed.* **131**, 15057-15061 (2019)

35 Lin, J., Li, G., She, C., Zhang, Y., Liu, S., Jing, C., ... & Chu, J. Microchannel tube NH_3 sensor based on metal-organic framework UiO-66 modified polyaniline. *Mater. Res. Bull.* **150**, 111770 (2022).

36 Bhardwaj, S. K., Mohanta, G. C., Sharma, A. L., Kim, K. H., & Deep, A. A three-phase copper MOF-graphene-polyaniline composite for effective sensing of ammonia. *Anal. Chim. Acta.* **1043**, 89-97 (2018).

37 Khan, F. U., Mehmood, S., Zhao, X., Yang, Y., Pan, X. presented at Abbrev. In 2021 IEEE International Symposium on Circuits and Systems, Daegu. Korea, May, (2021).

38 Wang, B., Li, H., Tan, H., Gu, Y., Chen, L., Ji, L., ... & Zhu, H. Gate-Modulated High-Response Field-Effect Transistor-Type Gas Sensor Based on the MoS2/Metal–Organic Framework Heterostructure. *ACS Appl. Mater. Interfaces*. **14**, 42356-42364 (2022).

39 Ko, M., Aykanat, A., Smith, M. K., & Mirica, K. A. Drawing Sensors with Ball-Milled Blends of Metal-Organic Frameworks and Graphite. *Sensors*. **17**, 2192 (2017).

40 Travlou, N. A., Singh, K., Rodriguez-Castellon, E., & Bandosz, T. J. Cu–BTC MOF–graphene-based hybrid materials as low concentration ammonia sensors. *J. Mater. Chem. A*. **3**, 11417-11429 (2015).

41 Yang, Y., Zhang, G., Luo, H., Yao, J., Liu, Z., & Zhang, D. Highly Sensitive Thin-Film Field-Effect Transistor Sensor for Ammonia with the DPP-Bithiophene Conjugated Polymer Entailing Thermally Cleavable tert-Butoxy Groups in the Side Chains. *ACS Appl. Mater. Interfaces*. **8**, 3635-3643 (2016).

42 Xue, M., Li, F., Chen, D., Yang, Z., Wang, X., & Ji, J. High-Oriented Polypyrrole Nanotubes for Next-Generation Gas Sensor. *Adv. Mater*. **28**, 8265-8270 (2016).

43 Jeevitha, G., Abhinayaa, R., Mangalaraj, D., Ponpandian, N., Meena, P., Mounasamy, V., & Madanagurusamy, S. Porous reduced graphene oxide (rGO)/WO_3 nanocomposites for the enhanced detection of NH_3 at room temperature. *Nanoscale Advances*. **1**, 1799-1811 (2019).

44 Mani, G. K., & Rayappan, J. B. B. Selective detection of ammonia using spray pyrolysis deposited pure and nickel doped ZnO thin films. *Appl. Surf. Sci.* **311**, 405-412 (2014).

45 Sun, Z., Huang, D., Yang, Z., Li, X., Hu, N., Yang, C., Yin, G. L., He, D. N., Zhang, Y. *IEEE electron device Lett*. **36**, 1376 (2015).

46 Lingling, W., Hui, H., Songhua, X., Daoping, C., Yuan, L., Bin, L., ... & Taihong, W. Enhanced sensitivity and stability of room-temperature NH_3 sensors using core-shell CeO_2 nanoparticles@cross-linked PANI with p-n heterojunctions. *ACS Appl. Mater. Interfaces*. **6**, 14131-14140 (2014).

47 Zhang, D., & Jiang, C. Room-temperature high-performance ammonia gas sensor based on layer-by-layer self-assembled molybdenum disulfide/zinc oxide nanocomposite film. *J. Alloys Compd.* **2017**, *698*, 476-483.

48 Dong, X., Cheng, X., Zhang, X., Sui, L., Xu, Y., Gao, S., ... & Huo, L. A novel coral-shaped Dy_2O_3 gas sensor for high sensitivity NH_3 detection at room temperature. *Sens. Actuators B Chem*. **255**, 1308-1315. (2018).

49 Abdulla, S., Mathew, T. L., & Pullithadathil, B. Highly sensitive, room temperature gas sensor based on polyaniline-multiwalled carbon nanotubes (PANI/MWCNTs) nanocomposite for trace-level ammonia detection. *Sens. Actuators B Chem*. **221**, 1523-1534 (2015).

50 Cui, S., Mao, S., Wen, Z., Chang, J., Zhang, Y., & Chen, J. Controllable synthesis of silver nanoparticle-decorated reduced graphene oxide hybrids for ammonia detection. *Analyst*. **138**, 2877-2882 (2013).

51 Chen, Y., Zhang, W., & Wu, Q. A highly sensitive room-temperature sensing material for NH_3: SnO2-nanorods coupled by rGO. *Sens. Actuators B Chem.* **242**, 1216-1226 (2017).

52 Jang, J., Chang, M., & Yoon, H. Chemical Sensors Based on Highly Conductive Poly(3,4-ethylenedioxythiophene) Nanorods. *Adv. Mater*. **17**, 1616-1620 (2005).

53 Sharma, S., Kumar, A., Singh, N., & Kaur, D. Excellent room temperature ammonia gas sensing properties of n-MoS_2/p-CuO heterojunction nanoworms. *Sens. Actuators B Chem*. **275**, 499-507 (2018).

54 Kumar, R., Kumar, R., Kushwaha, N., & Mittal, J. Ammonia Gas Sensing Using Thin Film of MnO_2 Nanofibers. *IEEE Sens.* J. **16**, 4691-4685 (2016).

55 Zhou, Y., Wang, J., & Li, X. Flexible room-temperature gas sensor based on poly (para-phenylene terephthalamide) fibers substrate coupled with composite NiO@CuO sensing materials for ammonia detection. *Ceram. Int*. **46**, 13827-13834 (2020).

56 Zhang, D., Jiang, C., Li, P., & Sun, Y. E. Layer-by-Layer Self-assembly of Co_3O_4 Nanorod-Decorated MoS2 Nanosheet-Based Nanocomposite toward High-Performance Ammonia Detection. *ACS Appl. Mater. Interfaces*. **9**, 6462-6471 (2017).

57 Lee, K., Gatensby, R., McEvoy, N., Hallam, T., & Duesberg, G. S. High-performance sensors based on molybdenum disulfide thin films. *Adv. Mater*. **25**, 6699-6702 (2013).

58 O'Brien, M., Lee, K., Morrish, R., Berner, N. C., McEvoy, N., Wolden, C. A., & Duesberg, G. S. Plasma assisted synthesis of WS_2 for gas sensing applications. *Chem. Phys. Lett.* **615**, 6-10 (2014).

59 Qin, Z., Ouyang, C., Zhang, J., Wan, L., Wang, S., Xie, C., & Zeng, D. 2D WS_2 nanosheets with TiO_2 quantum dots decoration for high-performance ammonia gas sensing at room temperature. *Sens. Actuators B Chem*. **253**, 1034-1042 (2017).

60 Rigoni, F., Tognolini, S., Borghetti, P., Drera, G., Pagliara, S., Goldoni, A., & Sangaletti, L. Enhancing the sensitivity of chemiresistor gas sensors based on pristine carbon nanotubes to detect low-ppb ammonia concentrations in the environment. *Analyst*. **138**, 7392-7399 (2013).

61 Shafiei, M., Hoshyargar, F., Lipton-Duffin, J., Piloto, C., Motta, N., & O'Mullane, A. P. Conversion of n-Type CuTCNQ into p-Type Nitrogen-Doped CuO and the Implication for Room-Temperature Gas Sensing. *J. Phys. Chem. C*. **119**, 22208–22216 (2015).

62 Panes-Ruiz, L. A., Shaygan, M., Fu, Y., Liu, Y., Khavrus, V., Oswald, S., ... & Cuniberti, G. Toward Highly Sensitive and Energy Efficient Ammonia Gas Detection with Modified Single-Walled Carbon Nanotubes at Room Temperature. *ACS Sens*. **3**, 79-86 (2018)

63 Kim, S. J., Koh, H. J., Ren, C. E., Kwon, O., Maleski, K., Cho, S. Y., ... & Jung, H. T. Metallic Ti_3C_2Tx MXene Gas Sensors with Ultrahigh Signal-to-Noise Ratio. *ACS nano*. **12**, 986-993 (2018).

64 Yavari, F., Chen, Z., Thomas, A. V., Ren, W., Cheng, H. M., & Koratkar, N. High sensitivity gas detection using a macroscopic three-dimensional graphene foam network. *Sci. Rep.*, **1**, 1-5 (2011).

65 Paul, R. K., Badhulika, S., Saucedo, N. M., & Mulchandani, A. Graphene Nanomesh As Highly Sensitive Chemiresistor Gas Sensor. *Anal. Chem*. **84**, 8171-8178 (2012).

66 Travlou, N. A., Seredych, M., Rodríguez-Castellón, E., & Bandosz, T. J. Activated carbon-based gas sensors: effects of surface features on the sensing mechanism. *J. Mater. Chem. A*. **3**, 3821-3831 (2015).

67 Liu, Y., Wang, L., Wang, H., Xiong, M., Yang, T., & Zakharova, G. S. Highly sensitive and selective ammonia gas sensors based on PbS quantum dots/TiO_2 nanotube arrays at room temperature. *Sens. Actuators B Chem*. **236**, 529-536 (2016).
