# Supplementary material for: Fabrication of robust and cost-efficient Hoffmann-type MOF sensors for room temperature ammonia detection
Source: Nat Commun. 2023 Nov 9;14:7261. doi: 10.1038/s41467-023-42959-z (PMC10636145; doi:10.1038/s41467-023-42959-z)
Supplement: Supplementary file 3 — Description of Additional Supplementary Files [file 41467_2023_42959_MOESM3_ESM.pdf]

Description of Additional Supplementary Files

File Name: Supplementary Data 1
Description: a. The atomic coordinates of the optimized computational models of NiNi-Pyz.
b. The atomic coordinates of the optimized computational models of CoNi-Pyz.
