# Supplementary material for: Fabrication of robust and cost-efficient Hoffmann-type MOF sensors for room temperature ammonia detection
Source: Nat Commun. 2023 Nov 9;14:7261. doi: 10.1038/s41467-023-42959-z (PMC10636145; doi:10.1038/s41467-023-42959-z)
Supplement: Supplementary file 4 — Supplementary Data 1 [file 41467_2023_42959_MOESM4_ESM.docx]

**Supplementary Data 1- a.** **The atomic coordinates of the optimized computational models of NiNi-Pyz**

| **Atom_site_label** | **Atom_site_type_symbol** | **Atom_site_fract_x** | **Atom_site_fract_y** | **Atom_site_fract_z** | **Atom_site_U_iso_or_equiv** | **Atom_site_adp_type** | **Atom_site_occupancy** |
| --- | --- | --- | --- | --- | --- | --- | --- |
| Ni1 | Ni | -0.00000 | -0.00062 | 0.50000 | 0.01123 | Uani | 1.00 |
| Ni2 | Ni | 0.50000 | 0.49933 | 0.50000 | 0.00923 | Uani | 1.00 |
| N3 | N | 0.50000 | 0.50000 | 0.21406 | 0.01527 | Uani | 1.00 |
| N4 | N | 0.50000 | 0.50000 | 0.78594 | 0.01527 | Uani | 1.00 |
| N5 | N | 0.68669 | 0.68607 | 0.50000 | 0.01460 | Uani | 1.00 |
| C6 | C | 0.80360 | 0.80242 | 0.50000 | 0.01420 | Uani | 1.00 |
| N7 | N | 0.31347 | 0.31257 | 0.50000 | 0.01460 | Uani | 1.00 |
| C8 | C | 0.19649 | 0.19627 | 0.50000 | 0.01420 | Uani | 1.00 |
| N9 | N | 0.31331 | 0.68606 | 0.50000 | 0.01460 | Uani | 1.00 |
| C10 | C | 0.19640 | 0.80242 | 0.50000 | 0.01420 | Uani | 1.00 |
| N11 | N | 0.68653 | 0.31257 | 0.50000 | 0.01460 | Uani | 1.00 |
| C12 | C | 0.80351 | 0.19627 | 0.50000 | 0.01420 | Uani | 1.00 |
| C13 | C | 0.50000 | 0.34159 | 0.10336 | 0.02433 | Uani | 0.50 |
| H14 | H | 0.50000 | 0.20841 | 0.17233 | 0.02900 | Uiso | 0.50 |
| C15 | C | 0.50000 | 0.66051 | 0.10848 | 0.02433 | Uani | 0.50 |
| H16 | H | 0.50000 | 0.79281 | 0.17889 | 0.02900 | Uiso | 0.50 |
| C17 | C | 0.50000 | 0.34159 | 0.89664 | 0.02433 | Uani | 0.50 |
| H18 | H | 0.50000 | 0.20841 | 0.82767 | 0.02900 | Uiso | 0.50 |
| C19 | C | 0.50000 | 0.66051 | 0.89152 | 0.02433 | Uani | 0.50 |
| H20 | H | 0.50000 | 0.79281 | 0.82111 | 0.02900 | Uiso | 0.50 |

**Supplementary Data 1 - b.** **The atomic coordinates of the optimized computational models of CoNi-Pyz**

| **Atom_site_label** | **Atom_site_type_symbol** | **Atom_site_fract_x** | **Atom_site_fract_y** | **Atom_site_fract_z** | **Atom_site_U_iso_or_equiv** | **Atom_site_adp_type** | **Atom_site_occupancy** |
| --- | --- | --- | --- | --- | --- | --- | --- |
| Co1 | Co | -0.00000 | -0.00063 | 0.50000 | 0.01123 | Uani | 1.00 |
| Co2 | Co | 0.50000 | 0.49931 | 0.50000 | 0.00923 | Uani | 1.00 |
| N3 | N | 0.50000 | 0.50003 | 0.20964 | 0.01527 | Uani | 1.00 |
| N4 | N | 0.50000 | 0.50003 | 0.79036 | 0.01527 | Uani | 1.00 |
| N5 | N | 0.68848 | 0.68782 | 0.50000 | 0.01460 | Uani | 1.00 |
| C6 | C | 0.80345 | 0.80247 | 0.50000 | 0.01420 | Uani | 1.00 |
| N7 | N | 0.31168 | 0.31080 | 0.50000 | 0.01460 | Uani | 1.00 |
| C8 | C | 0.19665 | 0.19620 | 0.50000 | 0.01420 | Uani | 1.00 |
| N9 | N | 0.31152 | 0.68782 | 0.50000 | 0.01460 | Uani | 1.00 |
| C10 | C | 0.19655 | 0.80247 | 0.50000 | 0.01420 | Uani | 1.00 |
| N11 | N | 0.68832 | 0.31080 | 0.50000 | 0.01460 | Uani | 1.00 |
| C12 | C | 0.80335 | 0.19620 | 0.50000 | 0.01420 | Uani | 1.00 |
| C13 | C | 0.50000 | 0.34056 | 0.10213 | 0.02433 | Uani | 0.50 |
| H14 | H | 0.50000 | 0.20771 | 0.17212 | 0.02900 | Uiso | 0.50 |
| C15 | C | 0.50000 | 0.66157 | 0.10715 | 0.02433 | Uani | 0.50 |
| H16 | H | 0.50000 | 0.79351 | 0.17865 | 0.02900 | Uiso | 0.50 |
| C17 | C | 0.50000 | 0.34056 | 0.89787 | 0.02433 | Uani | 0.50 |
| H18 | H | 0.50000 | 0.20771 | 0.82788 | 0.02900 | Uiso | 0.50 |
| C19 | C | 0.50000 | 0.66157 | 0.89285 | 0.02433 | Uani | 0.50 |
| H20 | H | 0.50000 | 0.79351 | 0.82135 | 0.02900 | Uiso | 0.50 |
